# Surgical Treatment of Tuberculous Peritonitis

**Published:** 1890-12-13

**Authors:** 


### SURGICAL TREATMENT OF TUBERCULOUS PERITONITIS.

The brilliant results that have followed abdominal section in cases of tubercle of the peritoneum cannot be gainsaid, the only question is the way in which the cure is brought about. M. Maurange has collected statistics of 71 cases in which the operation had been performed. Twelve of these died soon after, but 30 were doing well one or more years afterwards, and even in those that succumbed to pulmonary tuberculosis later on the peritoneal disease was frequently found to be completely cured. M. Maurange attributes the arrest of the local affection to the thorough antisepsis and the removal of the serous effusion, which is an excellent cultivation fluid. He has, however, seen equally good results following a less serious procedure, viz., removal of the ascitic fluid by aspiration, washing out the abdominal cavity with some antiseptic solution, which is then aspirated out; and finally, the injection into the cavity of four grams of iodoform dissolved in 100 of liquid oil of vaseline. This injection may be safely repeated, since the dose of iodoform is but small, and the power of absorption possessed by the peritoneum is comparatively feeble.

